# Graft repair during machine perfusion: a current overview of strategies

**DOI:** 10.1097/MOT.0000000000001151

**Published:** 2024-05-10

**Authors:** Roberto Broere, Stefan H. Luijmes, Jeroen de Jonge, Robert J. Porte

**Affiliations:** Department of Surgery, Division of Hepato-Pancreato- Biliary and Transplant Surgery, Erasmus MC Transplant Institute, University Medical Center Rotterdam, Rotterdam, The Netherlands

**Keywords:** graft repair, liver transplantation, machine perfusion, regenerative medicine

## Abstract

**Purpose of review:**

With changing donor characteristics (advanced age, obesity), an increase in the use of extended criteria donor (ECD) livers in liver transplantation is seen. Machine perfusion allows graft viability assessment, but still many donor livers are considered nontransplantable. Besides being used as graft viability assessment tool, *ex situ* machine perfusion offers a platform for therapeutic strategies to ameliorate grafts prior to transplantation. This review describes the current landscape of graft repair during machine perfusion.

**Recent findings:**

Explored anti-inflammatory therapies, including inflammasome inhibitors, hemoabsorption, and cellular therapies mitigate the inflammatory response and improve hepatic function. Cholangiocyte organoids show promise in repairing the damaged biliary tree. Defatting during normothermic machine perfusion shows a reduction of steatosis and improved hepatobiliary function compared to nontreated livers. Uptake of RNA interference therapies during machine perfusion paves the way for an additional treatment modality.

**Summary:**

The possibility to repair injured donor livers during *ex situ* machine perfusion might increase the utilization of ECD-livers. Application of defatting agents is currently explored in clinical trials, whereas other therapeutics require further research or optimization before entering clinical research.

## INTRODUCTION

To date, liver transplantation remains the only curative option for patients with end-stage liver disease or selected malignancies, but the shortage of suitable donor organs remains a major hurdle to expand transplant activity. The use of extended criteria donor (ECD) livers may bridge the gap between available donor organs and donor livers needed. However, these ECD livers are associated with a higher incidence of posttransplant complications compared to standard grafts, as they are more susceptible to ischemia-reperfusion injury (IRI) [1–3].

IRI is a biphasic phenomenon, in which cellular damage following ischemia is accentuated upon reintroduction of blood supply in the recipient [4]. The release of reactive oxidative species (ROS) by mitochondria upon reperfusion leads to the release of inflammatory molecules, such as danger-associated molecular patterns [5–10]. This triggers a sterile inflammatory response due to the additional release of cytokines and recruitment of neutrophils into the donor graft [11–14].

*Ex situ* machine perfusion (MP) is a preservation technique based on the circulation of a perfusate through the donor organ. Oxygenated hypothermic machine perfusion (HMP) is performed at low temperatures and reduce IRI-associated inflammation by replenishing adenosine triphosphate (ATP)-levels and mitochondrial ROS production during graft reperfusion [7]. Normothermic machine perfusion (NMP) is performed at physiological temperatures (37°C), thereby allowing viability assessment, leading to increased utilization of ECD livers [15–18]. Nevertheless, up to one third of the tested livers do not meet viability criteria and are still discarded.

Donor organs that are too damaged for direct transplantation could theoretically be repaired outside the body with targeted interventions within the isolated environment of MP. Administered therapies are directly delivered to the organ, thereby minimizing the chances of unintended systemic adverse effects in the recipient. In this review, we will address proposed treatment modalities for injured donor livers during MP, from therapies that are at the start of possible clinical implementation to more futuristic concepts.

The current approaches to repair livers can be grouped in two main categories. One of these categories aims to reduce inflammation and cell injury through inflammasome inhibition, hemoabsorption, cellular therapies, defatting and RNA interference. The other category, focusing on the regeneration of tissue can be reached through organoid infusion and repair. These approaches are discussed in more detail below. 

**Box 1:**
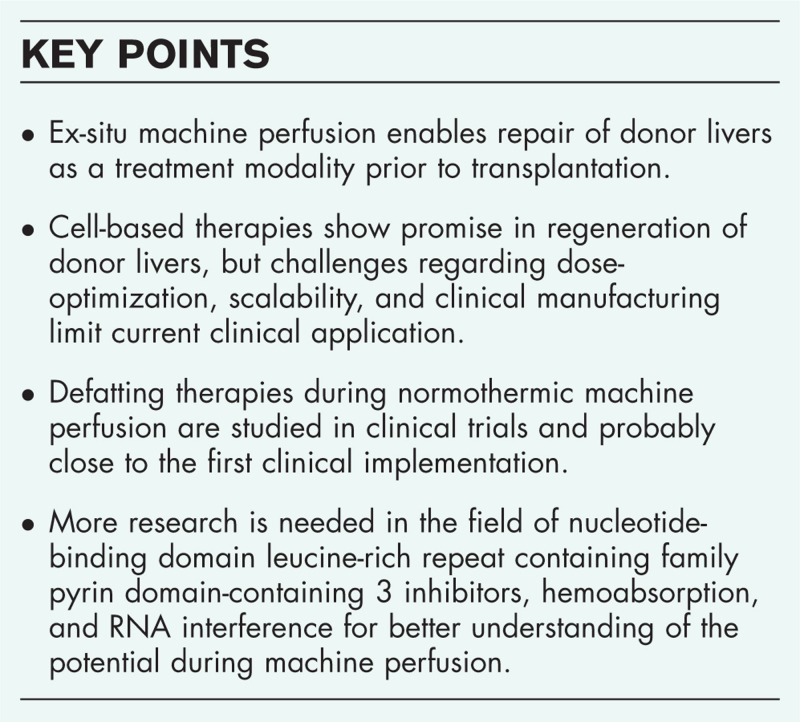
no caption available

## INFLAMMASOME INHIBITORS

The activation of the nucleotide-binding domain leucine-rich repeat containing family pyrin domain-containing 3 (NLRP3) inflammasome is a crucial event in the inflammatory cascade of IRI. Formation of the NLRP-3 inflammasome triggers downstream activation of caspases, leading to the secretion of interleukin (IL-)1β and IL-18, and ultimately resulting in pyroptotic cell death [19–23]. The effectiveness of MCC950 and Emricasan (MedChemExpress, USA), compounds inhibiting NLRP3 inflammasome activation, has been explored in two preclinical MP studies [24,25]. In a porcine transplantation model, administering MCC950 during HMP reduced the expression of pro-inflammatory cytokines tumor necrosis-alpha (TNF-α) and IL-1-β, attenuated Kupffer cell activation, and resulted in reduced histological liver damage posttransplantation [24]. Additionally, markers of liver injury such as aspartate aminotransferase (AST), total bilirubin, and malondialdehyde levels decreased significantly. Treatment of discarded human livers with Emricasan during NMP demonstrated a reduction in pro-inflammatory responses to IRI, as demonstrated by lower perfusate concentrations of IL-6, IL-8, and interferon (IFN)-γ [25]. Moreover, decreased levels of apoptotic cell death markers, including caspase-3/7 enzyme activity and cell-free DNA levels, were observed following Emricasan administration. However, it is important to note that the use of MCC950 was associated with drug-induced liver injury, whereas high dosages of Emricasan in the preservation fluids leaded to aggravation of ischemia-related inflammation after reperfusion in human transplantation [26,27]. Therefore, before clinical application of NLRP-3 inhibitors, dose-response studies are necessary to confirm safety and tolerability.

## HEMOABSORPTION

Hemoabsorbers consist of columns containing biocompatible, porous polymer sorbent beads with high binding capacity for circulating cytokines and inflammation mediators of varying molecular sizes. Cytosorb (CytoSorbents Corporation, USA), a commercially available hemoabsorber, is clinically applied for treatment of liver failure, septic shock, and vasoplegic shock [28–30]. Ghinolfi *et al.* investigated the feasibility and effects of incorporating a Cytosorb filter in the perfusion circuit during NMP in porcine liver grafts [31]. Lactate clearance was improved in livers treated with Cytosorb compared to controls, while no significant differences in alanine aminotransferase (ALT) and inflammatory cytokine concentrations were observed between the groups. This study consisted of a small sample size and more studies wither higher numbers are required to determine efficacy of Cytosorb treatment. Hemoabsorption filters remove inflammatory factors in a nonselective manner, potentially leading to a reduction in beneficial anti-inflammatory mediators and drugs [32,33]. Hence, the lack of selectivity and possible adverse effects warrant further investigation in preclinical perfusion models.

## CELLULAR AND CELL-DERIVED THERAPIES

Therapies with stromal cells, including mesenchymal stromal cells (MSC) and multi adult progenitor cells (MAPC), or cell-derived extracellular vesicles (EV) focus on modulation of the IRI-related inflammatory response. Cellular therapies, such as organoids, aim to repair damaged tissue by the replacement of healthy cells. Multiple studies have assessed the effects of these cellular and cell-derived therapies within the context of MP.

### Mesenchymal stromal cells

MSC exert anti-inflammatory and regenerative effects through interactions with immune cells and paracrine secretion of cytokines and growth factors [34,35]. Numerous rodent studies have investigated the effects of rat bone marrow-derived MSC administration during NMP in transplant models [36–42]. MSC injection resulted in decreased liver and biliary injury posttransplantation, reflected by lower levels of ALT, AST, gamma-glutamyl transferase, alkaline phosphatase, and total bilirubin [36–41]. Additionally, MSC injection ameliorated markers associated with oxidative stress, as seen by an increase in glutathione and a decrease in malondialdehyde, myeloperoxidase, and reactive oxidative species [38,41]. Also, MSC-treated livers showed improved mitochondrial function, demonstrated by a higher mitochondrial membrane potential [41].

Immunoregulatory effects of MSCs were demonstrated by reduced levels of anti-inflammatory cytokines, including IL-1-β, IL-2, IL-6, and TNF-α [36,37,40]. Histopathological analysis of MSC-treated livers showed decreased mitochondrial damage, less hepatocyte apoptosis, and decreased liver damage as assessed by the Suzuki-score [36–42]. Rodents that received MSC during NMP had a better prognosis and liver function, as seen by improved survival rates, higher bile production, and enhanced lactate clearance [36–42]. Treatment with MSC promoted the proliferation of residual peribiliary gland cells (PBGs), inhibited cell apoptosis and increased the proportion of pluripotent cells in PBGs, indicating repair of the biliary epithelium [39]. In a similar allogenic transplant model, MSC infusion during NMP alleviated the effects of acute rejection through inhibition of dendritic cell maturation and reduction of natural killer cells after transplantation [36,40]. In addition, treatment with MSC resulted in a lower rejection activation index and lower concentration of cytokines related to acute rejection.

The effects of MSC administration during liver MP have been explored in only one experimental study in pigs [43]. Verstegen *et al.* infused labeled fluorescent-labelled human MSC during HMP via either the portal vein or hepatic artery, followed by a short period of NMP [43]. Tracking of MSC revealed uniform distribution of infused cells within the liver during warm reperfusion and higher levels of anti-inflammatory cytokines IL-6 and IL-8 were measured in the perfusate.

MAPC are adult, bone marrow-derived stromal cells closely related to MSC [44]. Laing *et al.* showed that the injection of fluorescent-labelled MAPC in human discarded livers during NMP did not affect flow rates and resistance [45]. MAPC infused via the hepatic artery migrated across the vascular endothelium and homed into the liver parenchyma, whereas cells injected via the portal vein arrested in the sinusoidal space. Perfusate analysis revealed secretion of immunomodulatory factors by MAPC.

Application of MSC and MAPC during MP appeared to be safe and led to anti-inflammatory effects. However, the optimal dosage was not subject in any of the published studies. Therefore, dose response studies should be performed before clinical trials are initiated. Multiple commercial companies produce clinical-grade allogeneic MSC and MAPC. However, the use of autologous MSC necessitates in-house facilities compliant with good manufacturing practice (GMP) regulations.

### Human liver stem cell extracellular vesicles

EV represent a diverse array of cell-derived membranous structures crucial for intercellular communication [46–48]. They serve as carriers for the transfer of proteins, lipids, and genetic material, playing a pivotal role in cellular signaling. EV of human liver stem cells (HLSC-EV) have been administered in rat livers during NMP, resulting in lower concentrations of damage markers ALT, AST, and lactate dehydrogenase (LDH) [49,50]. Also, injection of HLSC-EVs led to improved liver function and metabolic activity, reflected by increased bile production, improved phosphate utilization, and enhanced pH regulation. Histological assessment of treated liver tissues demonstrated less apoptosis and necrosis of hepatocytes and lower Suzuki-scores compared to controls. EV represent an interesting alternative to cellular therapies, but issues with regard to standardized EV-manufacturing and non-GMP-compliance needs to be resolved prior to translation to clinical research and practice.

### Cholangiocyte organoids

Organoids are three-dimensional cell clusters that mimic the functionalities of the tissue of origin [51]. The proliferative and differentiation potential of cholangiocyte organoids holds promise to repair bile duct injury prior to transplantation. Administration of cholangiocyte organoids into the intrahepatic bile ducts of human discarded livers during NMP resulted in cellular engraftment and consequent regeneration of the biliary epithelium [52]. Injected organoids expressed region-specific biliary markers and retained function in modifying bile composition, reflected by increased bile pH and bile production. Organoids are typically cultured in non-GMP-compliant tumor-derived basement membrane extracts, necessitating the development of alternative extracellular matrix proteins before clinical use can be considered [51,53,54].

## DEFATTING STRATEGIES

In order to intervene successfully in fat metabolism, normothermic conditions are required in order to have a metabolically active organ [55,56]. In a study on porcine livers, NMP alone, without specific defatting agents, already decreased steatosis from 30% to 15% over 48 h [57]. However, when performing NMP in human livers, no significant reduction in steatosis was seen [58], despite increasing triglyceride (TG) levels in the perfusion fluid during 24-h of perfusion.

A frequently used “defatting cocktail” consists of forskolin, scoparone, visfatin, hypericin (PXR ligand), GW7647 (PPAR-α ligand), and GW501516 (PPAR-δ ligand) [59,60]. Sometimes, l-carnitinine is also added to the defatting cocktail [61,62].

In three MP studies with (fatty) livers from Zucker rats [59–61], the defatting cocktail increased TG in the perfusate, but did not consistently reduce intracellular TG or lipid droplets when performed during subnormothermic MP (20°C) [60]. However, when administrated during NMP, a 30% decrease in liver TG was observed [59]. Levels of ketones increased during NMP, suggesting increased lipid metabolism compared to a nontreated group, along with increased bile production [59]. Addition of L-carnitine to the defatting cocktail improved lactate clearance and hepatic function, as reflected in higher bicarbonate levels in bile [61]. However, in this particular study, no histological differences were seen between the two groups [61].

Other compounds, like glial cell line-derived neurotrophic factor (GDNF) alone [63] and a modified version of the defatting cocktail (replacing visfating and GW chemicals with epigallocatechin-3-gallate and resveratrol) [64], have been tested for defatting of livers. Both approaches showed a reduction in intrahepatic TG content in steatotic rodent livers during NMP. However, levels of LDH [63], ALT, and AST [64] were lower compared to the standard defatting cocktail, suggesting less hepatocyte injury.

(1) In the first study performing experimental defatting strategies in 2 macrosteatotic human discarded livers, l-carnitine and exendin-4 were added and led to a reduction of 10% in macrosteatosis after 8 h of NMP [65]. In another more detailed study, 10 discarded steatotic human livers were subjected to 12-h of NMP [62]. The treatment group received the standard defatting cocktail with the addition of l-carnitine, whereas the control group received NMP only. After 6 h, tissue TG in the treatment group decreased with 38% vs. 7% in the control group. Macrosteatosis decreased by 40% in 6 h, 50% in 12 h in the defatting group, whereas there were no changes seen in the control group. Lactate levels decreased significantly in the treatment group, with no changes in the control group. In addition, bile production and bile-pH improved after defatting. Perfusate ALT levels were lower after 12 h of NMP in the treatment group. Defatting resulted in less Kupffer cell and neutrophil activation, together with decreased levels of TNF-α and IL-1-β [62], indicating less inflammation. The Zurich group recently showed exciting results in a long-term (7–12 days) experimental NMP study on defatting human livers. Addition of L-carnitine and fenofibrate resulted in a decrease in tissue TGs, with some grafts losing nearly all TG seen in the liver biopsy prior to perfusion [66^▪▪^]. In livers successfully reacting to the defatting therapy, increased bile production and factor V synthesis was observed, suggesting better metabolic function [66^▪▪^]. Although NMP during 7 to 12 days is not yet clinically feasible, development of liver defatting during extended MP will likely become available in the near future.

One of the issues that need to be resolved is the availability of clinical grade defatting compounds. There are some concerns about GW compounds, that they might stimulate cell proliferation and are potentially liver carcinogenic, but this may not be relevant in short-term NMP [67,68]. Direct toxicity from defatting compounds has not been observed [69]. Alternatively, fat released during defatting on NMP can clog oxygenators and cause micro-embolisms; fat filters could help overcome this issue. A first step towards wider application is the NHS-BT DeFat trial, randomizing 60 steatotic liver grafts between 6 h of normal NMP or NMP supplemented with defatting agents, to assess viability criteria and organ utilization for transplantation (ISRCTN 14957538).

## RNA-INTERFERENCE

RNA inhibition (RNAi) was discovered using small RNA molecules binding to messenger RNA (mRNA) [70]. Administering small interfering RNA (siRNA) binds fully to its target mRNA, allowing very specific gene silencing. SiRNA can be chemically synthesized in high volumes for targeting specific mRNA [71,72].

In the first study with RNAi during MP, siRNA uptake by rat hepatocytes was demonstrated in HMP and NMP [73]. In a rat liver transplant study, siRNA against the Fas cell surface death receptor was tested during 1 h of HMP, showing discrete uptake by liver tissue. IL-10 (anti-inflammatory) levels increased postreperfusion, but no significant differences were observed in transaminase levels, necrosis, apoptosis, or FAS protein transcription between the intervention and control group [74^▪^]. The limited efficacy of siRNA during HMP may be due to using “naked” siRNA. Conjugating siRNA with cholesterol or delivering through lipid nanoparticles may enhance liver uptake and retention [75–77]. Generally, RNAi therapy relies on active metabolic conditions, and 1 h of HMP might not be sufficient for siRNA uptake and effect.

RNAi during MP is a relatively new area of research. While there are some initial indications of its feasibility, substantial therapeutic benefits for the liver have not yet been demonstrated.

## CONCLUSION

The use of ECD livers aims to expand the donor pool to reduce waiting list mortality. Technical advances in MP allows assessment of ECD liver viability, but one third of these organs is still considered unsuitable for transplantation. Therapies during NMP show promise in repairing injured livers by reducing inflammation, initiating repair, and enhancing liver function. In other words, making the unsuitable donor liver suitable for transplantation. Repairing organs outside the body was once futuristic, but with long-term NMP reaching up to 12 days, this may unlock a whole new era in regenerative medicine.

## Acknowledgements

**FIGURE 1:**
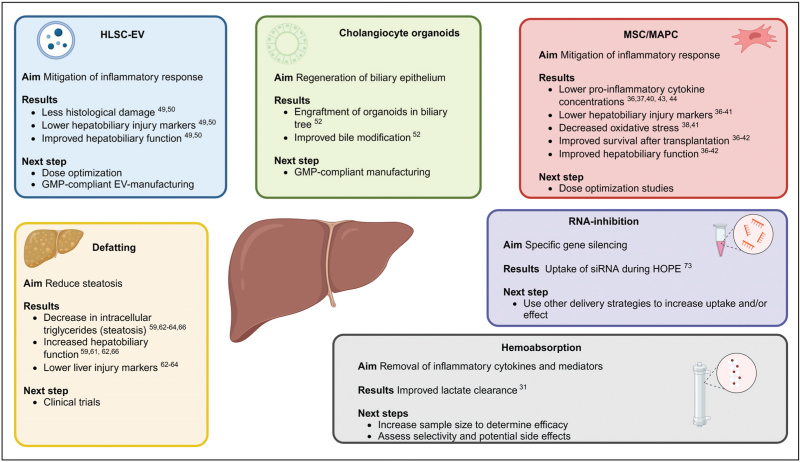
Therapeutic possibilities for repair and regeneration of donor livers during machine perfusion. A donor liver that is too injured for transplantation can be repaired by using several therapeutic strategies, including defatting agents, human liver stem cell extracellular vesicles (HLSC-EV), cholangiocyte organoids, mesenchymal stromal cells (MSC), multi adult progenitor cells (MAPC), RNA-interference therapy, and incorporation of hemoabsorbers. EV, extracellular vesicles; GMP, general manufacturing practice; HLSC, human liver stem cell extracellular vesicles; HOPE, hypothermic oxygenated perfusion; MAPC, multi adult progenitor cells; MSC, mesenchymal stromal cells; siRNA, small interfering RNA.

*We acknowledge Mrs. N. Delleman for the help with* *Fig. 1* *that was created with Biorender.com.*

### Financial support and sponsorship

*None.*

### Conflicts of interest

*There are no conflicts of interest.*

## REFERENCES AND RECOMMENDED READING

Papers of particular interest, published within the annual period of review, have been highlighted as:

▪ of special interest
▪▪ of outstanding interest
